# STEAP1 Knockdown Decreases the Sensitivity of Prostate Cancer Cells to Paclitaxel, Docetaxel and Cabazitaxel

**DOI:** 10.3390/ijms24076643

**Published:** 2023-04-02

**Authors:** Sandra M. Rocha, Daniel Nascimento, Rafaella S. Coelho, Ana Margarida Cardoso, Luís A. Passarinha, Sílvia Socorro, Cláudio J. Maia

**Affiliations:** 1 CICS-UBI–Health Sciences Research Center, Universidade da Beira Interior, 6201-506 Covilhã, Portugal; sandra.rocha@ubi.pt (S.M.R.);; 2 Associate Laboratory i4HB-Institute for Health and Bioeconomy, NOVA School of Science and Technology, Universidade NOVA de Lisboa, 2819-516 Caparica, Portugal; 3 UCIBIO–Applied Molecular Biosciences Unit, Department of Chemistry, NOVA School of Science and Technology, Universidade NOVA de Lisboa, 2819-516 Caparica, Portugal; 4 Laboratório de Fármaco-Toxicologia-UBIMedical, Universidade da Beira Interior, 6201-284 Covilhã, Portugal; 5 C4-UBI—Cloud Computing Competence Center, Universidade da Beira Interior, 6200-501 Covilhã, Portugal

**Keywords:** prostate cancer, paclitaxel, docetaxel, cabazitaxel, STEAP1

## Abstract

The Six Transmembrane Epithelial Antigen of the Prostate 1 (STEAP1) protein has been indicated as an overexpressed oncoprotein in prostate cancer (PCa), associated with tumor progression and aggressiveness. Taxane-based antineoplastic drugs such as paclitaxel, docetaxel, or cabazitaxel, have been investigated in PCa treatment, namely for the development of combined therapies with the improvement of therapeutic effectiveness. This study aimed to evaluate the expression of STEAP1 in response to taxane-based drugs and assess whether the sensitivity of PCa cells to treatment with paclitaxel, docetaxel, or cabazitaxel may change when the *STEAP1* gene is silenced. Thus, wild-type and STEAP1 knockdown LNCaP and C4-2B cells were exposed to paclitaxel, docetaxel or cabazitaxel, and STEAP1 expression, cell viability, and survival pathways were evaluated. The results obtained showed that STEAP1 knockdown or taxane-based drugs treatment significantly reduced the viability and survival of PCa cells. Relatively to the expression of proliferation markers and apoptosis regulators, LNCaP cells showed a reduced proliferation, whereas apoptosis was increased. However, the effect of paclitaxel, docetaxel, or cabazitaxel treatment was reversed when combined with STEAP1 knockdown. Besides, these chemotherapeutic drugs may stimulate the cell growth of PCa cells knocked down for STEAP1. In conclusion, this study demonstrated that STEAP1 expression levels might influence the response of PCa cells to chemotherapeutics drugs, indicating that the use of paclitaxel, docetaxel, or cabazitaxel may lead to harmful effects in PCa cells with decreased expression of STEAP1.

## 1. Introduction

The Six-Transmembrane Epithelial Antigen of the Prostate (STEAP1) protein has been identified as being upregulated in several human cancers, with emphasis on prostate cancer (PCa) [1,2,3,4,5,6]. STEAP1 acts as a metalloreductase contributing to the generation of reactive oxygen species, which induces intracellular oxidative stress and inflammation [7,8,9,10]. Several researchers exploring the role of STEAP1 in cancer have shown that its overexpression inhibits apoptosis, enhances cell proliferation, migration, and invasion, and induces epithelial to mesenchymal transition, ultimately contributing to tumor progression and aggressiveness [11,12,13,14,15,16]. Moreover, STEAP1 expression levels in human PCa were reported to be 5- to 10-fold higher compared to other cancer types [1]. Due to its high tumor specificity and membrane-bound localization, STEAP1 is currently considered an oncogene and a promising therapeutic target for PCa [6,17,18]. 

Chemotherapy is a type of treatment often used to treat cancer cells, which utilizes powerful chemicals to kill fast-growing cells [19,20]. Chemotherapeutic drugs can be used alone or in combination with other types of treatments to treat a wide variety of cancers. Different chemotherapeutic agents with distinct mechanisms of action are available [19], which include taxanes, an important class of anti-microtubule agents [21]. Taxanes exert anti-cancer effects by binding tubulin and affecting microtubule polymerization, which results in mitotic arrest and induction of apoptosis in highly proliferating cancer cells [22]. Paclitaxel was the first taxane to receive regulatory approval for use as anti-cancer therapy in the United States [23]. Later, docetaxel was produced as a second-generation semisynthetic taxane analogue with better tolerability and cytotoxicity [24]. Cabazitaxel is a novel third-generation semisynthetic analogue of docetaxel, which was investigated as a promising agent for the treatment of castration-resistant PCa (CRPC) [25]. In the last years, several studies have shown that cabazitaxel is effective in improving the life quality of CRPC patients [25,26,27,28]. However, the clinical benefit of these taxane-based chemotherapeutics administration is limited in CRPC treatment, showing a modest effect on patient survival, triggering toxicity in normal tissues, and some studies have even reported the death of patients [29,30]. Therefore, it is crucial to evaluate the effect of chemotherapeutic drugs modulating the mechanisms of cell cycle control, namely the expression of oncogenes, to define better treatment protocols and the effectiveness of combined therapies for PCa treatment. Also, it is critical to understand if manipulating oncogenes expression can impact the response to chemotherapeutics.

The present study aimed to evaluate the expression of STEAP1 in response to taxane-based drugs, and to determine if the sensitivity of PCa cells to treatment with chemotherapeutic drugs changes when *STEAP1* gene is knocked down. For this purpose, human neoplastic cells with different levels of STEAP1 expression were exposed to paclitaxel, docetaxel or cabazitaxel. Alterations in cell viability, proliferation, and apoptosis, as well as in the expression of STEAP1 and target regulators of cell proliferation and apoptosis, were assessed. 

## 2. Results

### 2.1. Effect of Paclitaxel, Docetaxel and Cabazitaxel on STEAP1 Expression in PCa Cells

**Figure 1:**
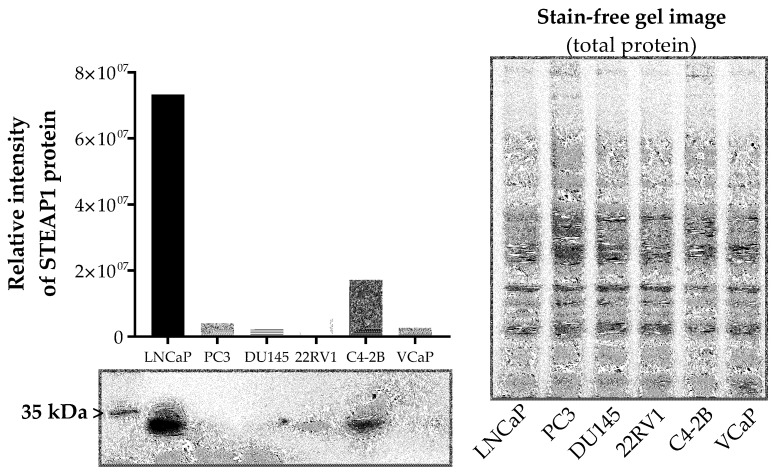
Comparison of STEAP1 expression levels in different PCa cell lines. Relative immunoreactivity of STEAP1 in PCa cell lines (LNCaP, PC3, DU145, 22RV1, C4-2B and VCaP) by Western blot.

To investigate the biological role of STEAP1 related to the chemotherapy of PCa, the protein levels of STEAP1 in different PCa cell lines (LNCaP, PC3, DU145, 22RV1, C4-2B and VCaP) were quantified by Western blot. The results showed that LNCaP and C4-2B cells express the STEAP1 protein (Figure 1), being even higher in LNCaP cells.

**Figure 2:**
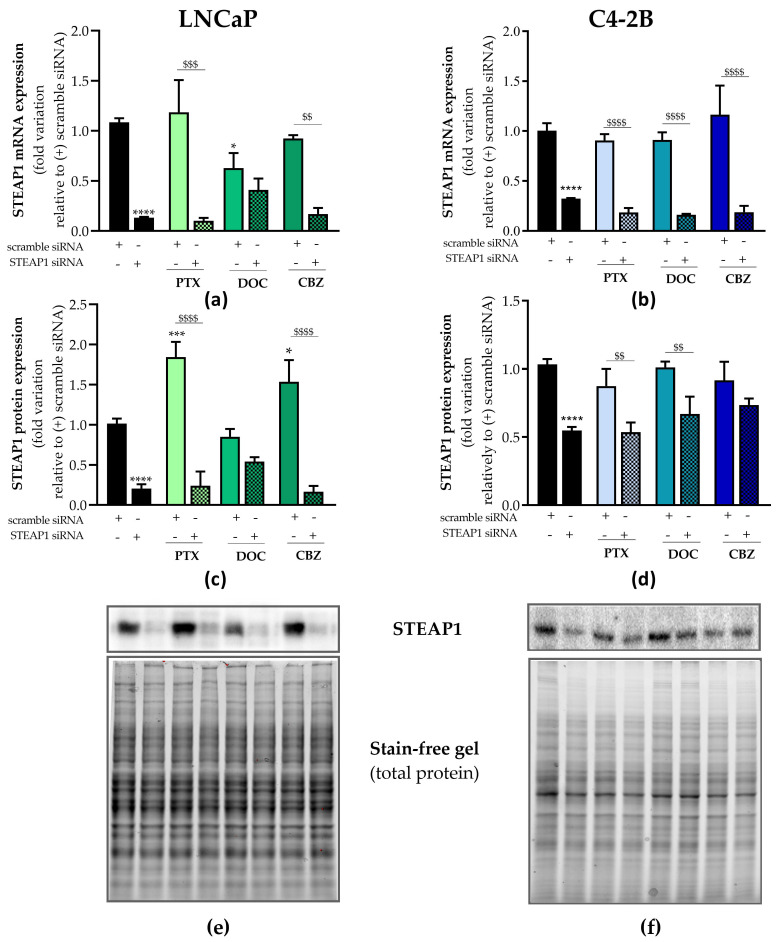
STEAP1 silencing in LNCaP and C4-2B cells and effect of taxane-based drugs on STEAP1 expression. Human neoplastic LNCaP and C4-2B prostate cells were transfected with scramble or STEAP1 small interfering RNA (siRNA) for 24 h and treated with 5 nM paclitaxel (PTX), 20 nM docetaxel (DOC) or 1 nM cabazitaxel (CBZ) for an additional 24 h. (**a**,**b**) Relative STEAP1 mRNA expression determined by RT-qPCR after normalization with the β2M housekeeping gene. (**c**,**d**) Relative STEAP1 protein expression determined by Western blot analysis after normalization with total protein. (**e**,**f**) SDS-PAGE gels’ representative image. The symbol “+” or “-“means presence or absence of the types of siRNA used in each experimental group. Results are represented as fold-variation in comparison to scramble siRNA (control group). Error bars show mean ± S.E.M (n ≥ 3). * *p* < 0.05, *** *p* < 0.001 and **** *p* < 0.0001 in comparison with the scramble siRNA group; and ^$$^ *p* < 0.01, ^$$$^ *p* < 0.001 and ^$$$$^ *p* < 0.0001.

The silencing of the *STEAP1* gene in LNCaP and C4-2B cells was performed, and the effect of chemotherapeutics paclitaxel, docetaxel, or cabazitaxel in restoring STEAP1 expression was evaluated. The levels of STEAP1 mRNA and protein were determined by RT-qPCR and Western blot, respectively. As indicated in Figure 2, the STEAP1 mRNA and protein levels were significantly diminished in the STEAP1 siRNA group of both cell lines (87 ± 0.01% and 80 ± 0.05% reduction for mRNA and protein, respectively, to LNCaP cells; 68 ± 0.003% and 45 ± 0.01% reduction for mRNA and protein, respectively, to C4-2B cells) compared to scramble siRNA. 

LNCaP and C4-2B cells transfected with scramble siRNA or STEAP1 siRNA were treated with 5 nM paclitaxel, 20 nM docetaxel or 1 nM cabazitaxel for 24 h. No significant differences were observed in STEAP1 mRNA expression of LNCaP cells with normal STEAP1 expression levels (scramble siRNA group) upon treatment with paclitaxel or cabazitaxel treatment (Figure 2a). However, paclitaxel and cabazitaxel induced a significant increase in STEAP1 protein expression compared with the scramble siRNA condition (1.845 ± 0.19- vs. 1.016 ± 0.06- and 1.536 ± 0.27- vs. 1.016 ± 0.06-fold variation, respectively, Figure 2c). In opposition, docetaxel treatment significantly decreased STEAP1 mRNA levels in LNCaP cells transfected with scramble siRNA (0.629 ± 0.15- vs. 1.085 ± 0.04-fold variation, Figure 2a). Relatively to C4-2B cells, no significant differences were observed in STEAP1 mRNA and protein expression upon treatment with paclitaxel, docetaxel and cabazitaxel in scramble siRNA or STEAP1 siRNA conditions (Figure 2b and 2d). 

None of the tested chemotherapeutics drugs altered the effect of silencing STEAP1 in LNCaP and C4-2B cells, concerning the expression of STEAP1 mRNA and protein (Figure 2). 

### 2.2. Effect of STEAP1 Gene Knockdown Associated with Taxane-Based Drugs on PCa Cells Viability 

**Figure 3:**
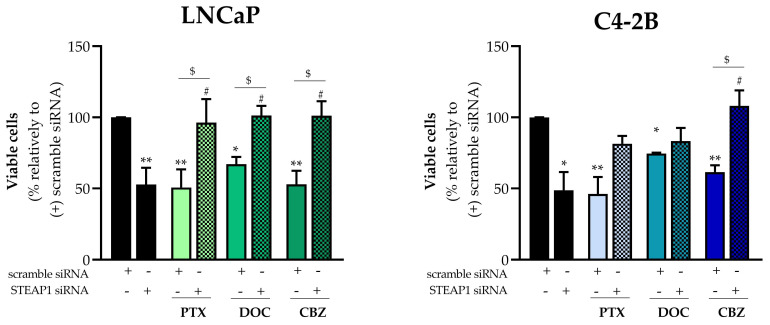
Effect of STEAP1 knockdown and paclitaxel, docetaxel, or cabazitaxel treatment on PCa cell viability. Human neoplastic LNCaP and C4-2B prostate cells were transfected with scramble or STEAP1 small interfering RNA (siRNA) for 24 h, and treated with 5 nM paclitaxel (PTX), 20 nM docetaxel (DOC) or 1 nM cabazitaxel (CBZ) for an additional 24 h. The symbol “+”or “-“means presence or absence of the type of siRNA used in each experimental group. The percentage of LNCaP and C4-2B viable cells was determined by MTT assay. Results are expressed as fold variation relative to the scramble siRNA group (control condition). Error bars show mean ± S.E.M (n ≥ 2). * *p* < 0.05 and ** *p* < 0.01 in comparison to the scramble siRNA group; ^#^ *p* < 0.05 when compared with the STEAP1 siRNA group; and ^$^ *p* < 0.05.

The viability of scramble or STEAP1 siRNA-transfected PCa cells after treatment with paclitaxel docetaxel or cabazitaxel was determined by the MTT assay. STEAP1-knockdown diminished the viability of LNCaP and C4-2B cells by 47.2 ± 11.8% and 48.7 ± 12.9%, respectively (Figure 3). Also, paclitaxel (5 nM), docetaxel (20 nM) and cabazitaxel (1 nM) significantly decreased the viability of mock-transfected (scramble siRNA) LNCaP cells (49.33 ± 12.8%, 32.97 ± 5.1% and 47.06 ± 9.5%, respectively) and C4-2B cells (46.2 ± 11.9%, 74.6 ± 0.6% and 61.4 ± 4.9%, respectively), compared to scramble siRNA control (Figure 3). Paclitaxel-, docetaxel-, and cabazitaxel-treated LNCaP cells knocked down for STEAP1 exhibited approximately two-fold higher viability than mock-transfected LNCaP cells treated with chemotherapeutic drugs (represented with ^$^, Figure 3). In addition, these drugs stimulated the cell viability in LNCaP cells knocked down for STEAP1 when compared to the respective control group (represented with ^#^, Figure 3). Concerning C4-2B cells, a similar effect was only observed with the cabazitaxel treatment.

### 2.3. Effect of STEAP1 Knockdown and Chemotherapeutic Drugs in Survival Pathways 

**Figure 4:**
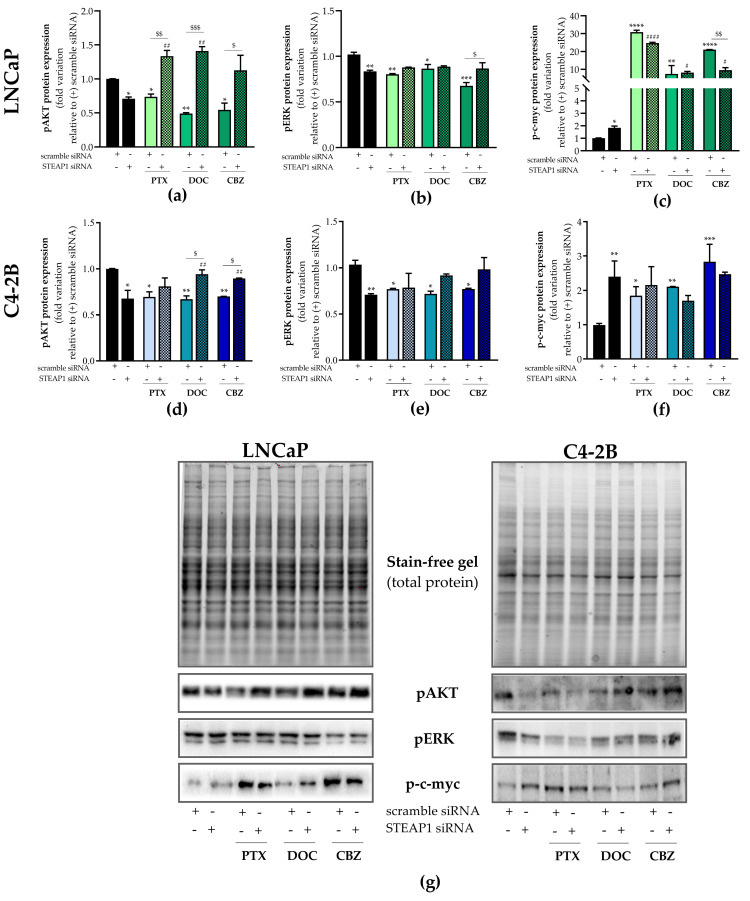
The effect of STEAP1 knockdown and paclitaxel, docetaxel, or cabazitaxel on the protein expression levels of the survival pathway regulators of PCa cells. Human neoplastic LNCaP and C4-2B prostate cells were transfected with scramble or STEAP1 small interfering RNA (siRNA) for 24 h and treated with 5 nM paclitaxel (PTX), 20 nM docetaxel (DOC) or 1 nM cabazitaxel (CBZ) for an additional 24 h. The symbol “+”or “-“means presence or absence of the type of siRNA used in each experimental group. Expression of (**a**,**d**) phosphorylated AKT, (**b**,**e**) ERK and (**c**,**f**) c-myc proteins was determined by Western blot analysis after normalization with total protein. (**g**) SDS-PAGE gel and representative immunoblots. Error bars indicate mean ± S.E.M (n ≥ 2). * *p* < 0.05, ** *p* < 0.01, *** *p* < 0.001 and **** *p* < 0.0001 when compared with the scramble siRNA group; ^#^ *p* < 0.05, ^##^ *p* < 0.01 and ^####^ *p* < 0.0001 when compared with the STEAP1 siRNA group; and ^$^ *p* < 0.05, ^$$^ *p* < 0.01 and ^$$$^ *p* < 0.001.

To better understand how STEAP1 knockdown reduces PCa cell viability and suppresses the effect of taxane-based drugs, the expression of target proteins associated with cell survival pathways was evaluated. The results of Western blot analysis demonstrated that the expression of phosphorylated-AKT (pAKT) and -ERK (pERK) isoforms decreased in LNCaP and C4-2B cells knocked down for STEAP1 relative to the scramble siRNA transfected cells (0.709 ± 0.02- vs. 1.001 ± 0.002-fold variation and 0.834 ± 0.01- vs. 1.02 ± 0.024-fold variation, respectively to LNCaP cells, Figure 4a and 4b; 0.678 ± 0.05- vs. 1.009 ± 0.003-fold variation and 0.710 ± 0.006- vs. 1.034 ± 0.03-fold variation, respectively to C4-2B cells, Figure 4d and 4e). Treatment of scramble siRNA transfected-cells with 5 nM paclitaxel, 20 nM docetaxel and 1 nM cabazitaxel significantly decreased pAKT in LNCaP cells (0.738 ± 0.04- vs. 1.001 ± 0.002-, 0.490 ± 0.01- vs. 1.001 ± 0.002-, and 0.546 ± 0.10- vs. 1.001 ± 0.002-fold variation, respectively, Figure 4a) and in C4-2B cells (0.695 ± 0.03- vs. 1.009 ± 0.003-, 0.670 ± 0.03- vs. 1.009 ± 0.003-, and 0.7 ± 0.002- vs. 1.009 ± 0.003-fold variation, respectively, Figure 4d). The same treatment also significantly decreased pERK in LNCaP cells (0.804 ± 0.01- vs. 1.020 ± 0.02-, 0.865 ± 0.04- vs. 1.020 ± 0.02-, and 0.677 ± 0.04- vs. 1.020 ± 0.02-fold variation, respectively, Figure 4b) and in C4-2B cells (0.771 ± 0.005- vs. 1.034 ± 0.03-, 0.720 ± 0.02- vs. 1.034 ± 0.03-, and 0.772 ± 0.005- vs. 1.034 ± 0.03-fold variation, respectively, Figure 4e) relative to the scramble siRNA control group. However, the silencing of STEAP1 in LNCaP cells significantly abolished the effect of paclitaxel, docetaxel, and cabazitaxel in suppressing pAKT. Furthermore, it should be highlighted that chemotherapeutic drugs increased the pAKT expression two-fold when the *STEAP1* gene was knocked down in LNCaP cells (Figure 4a). Regarding pERK expression levels in LNCaP cells, the silencing of STEAP1 did not reverse the effect of paclitaxel or docetaxel, whereas the down-regulation of pERK in cabazitaxel-treated LNCaP cells knocked down for STEAP1, was reversed (0.677 ± 0.05- vs. 0.867 ± 0.06-fold variation, Figure 4b). Relatively to C4-2B cells, the silencing of STEAP1 also significantly reversed the effect of docetaxel and cabazitaxel in pAKT expression (0.67 ± 0.03- vs. 0.941 ± 0.03-, and 0.7 ± 0.002- vs. 0.896 ± 0.003-fold variation, respectively, Figure 4d). Also, docetaxel or cabazitaxel induced a significantly increased expression of pAKT in C4-2B cells knocked down for STEAP1.

Regarding the c-myc transcription factor, the levels of phosphorylated c-myc (p-c-myc) were significantly increased in LNCaP and C4-2B cells upon silencing the *STEAP1* gene. p-c-myc expression in the STEAP1 siRNA group when compared to the scramble siRNA control was 1.846 ± 0.13- vs. 1.000 ± 0.03-fold variation and 2.401 ± 0.323- vs. 0.993 ± 0.02-fold variation (Figure 4c and 4f, respectively). Treatment of scramble siRNA mock-transfected LNCaP cells with paclitaxel, docetaxel, and cabazitaxel strongly induced the expression of p-c-myc (30.848 ± 1.07- vs. 1.000 ± 0.03-, 7.401 ± 4.67- vs. 1.000 ± 0.03-, and 21.077 ± 0.06- vs. 1.000 ± 0.03-fold variation, respectively, Figure 4c). The silencing of STEAP1 in LNCaP cells significantly reduced the cabazitaxel-induced p-c-myc expression (9.508 ± 1.440- vs. 21.077 ± 0.06-fold variation, Figure 4c), but no differences were found in the response to paclitaxel or docetaxel (Figure 4c). However, the expression of p-c-myc was significantly induced by paclitaxel, docetaxel, or cabazitaxel in LNCaP cells silenced for STEAP1 when compared to LNCaP cells silenced for STEAP1 (Figure 4c). Concerning C4-2B cells, the effect was similar to that observed in LNCaP cells but not so markedly (Figure 4f). Since the results obtained in LNCaP and C4-2B cells are overall similar in all experiments, LNCaP cells were chosen to explore the proliferative and apoptotic pathways underlying the effects of chemotherapeutic drugs when the *STEAP1* gene is knocked down.

### 2.4. Effect of STEAP1 Knockdown and Chemotherapeutic Drugs in Proliferative Activity

**Figure 5:**
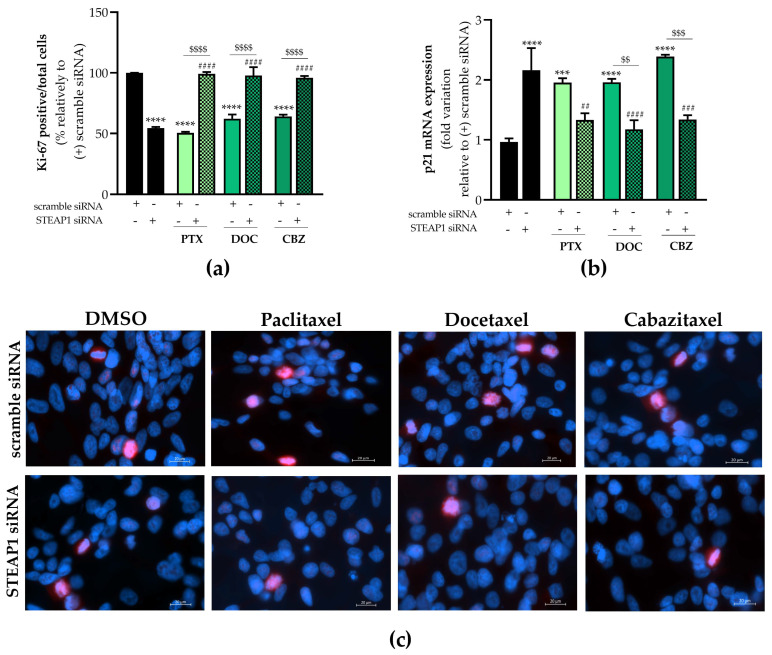
Effect of STEAP1 knockdown and paclitaxel, docetaxel or cabazitaxel on the proliferation activity on LNCaP cells. Human neoplastic LNCaP prostate cells were transfected with scramble or STEAP1 small interfering RNA (siRNA) for 24 h and treated with 5 nM paclitaxel (PTX), 20 nM docetaxel (DOC) or 1 nM cabazitaxel (CBZ) for an additional 24 h. The symbol “+” and “-” means presence or absence of the type of siRNA used in each experimental group. (**a**) Results of Ki-67 immunofluorescence analysis, representing the number of Ki-67-positive cells out of total cell number. Expression of (**b**) p21 mRNA; expression levels were determined by RT-qPCR analysis after normalization with β2M housekeeping gene. (**c**) Representative fluorescent immunocytochemistry images of Ki-67-labeled cells (red) and Hoechst 33342 stained nuclei (blue) obtained in the AxioImager Z2 microscope under 400× magnification. Ten randomly selected fields per microscope cover glass were assessed. Results are expressed as percentage or fold-variation relative to scramble siRNA (control group). Error bars indicate mean ± S.E.M (n ≥ 2). *** *p* < 0.001 and **** *p* < 0.0001 when compared with the scramble siRNA group; ^##^ *p* < 0.01, ^###^ *p* < 0.001 and ^####^ *p* < 0.0001 when compared with the STEAP1 siRNA group; and ^$$^ *p* < 0.01, ^$$$^ *p* < 0.001 and ^$$$$^ *p* < 0.0001.

Immunofluorescence staining of the nuclear proliferation marker ki-67 was used to evaluate the proliferation of LNCaP cells in different experimental conditions. The number of Ki-67-stained cells was significantly decreased in LNCaP cells knocked down for STEAP1, being 54.5 ± 1.01% when compared to scramble siRNA transfected cells (Figure 5a). Also, the results of fluorescent immunocytochemistry showed that the number of Ki-67-positive cells was significantly decreased in scramble siRNA-transfected LNCaP cells after treatment with 5 nM paclitaxel, 20 nM docetaxel or 1 nM cabazitaxel (50.5 ± 0.91%, 62.2 ± 3.6%, and 64.0 ± 1.54%, respectively, Figure 5a). The inhibitory effect on cell proliferation caused by paclitaxel, docetaxel, and cabazitaxel treatment was reversed in LNCaP cells knocked down for STEAP1. In addition, the chemotherapeutic drugs increased the number of Ki-67-positive cells in LNCaP cells knocked down for STEAP1 relative to LNCaP cells silenced for STEAP1 (Figure 5a). The p21 protein, a well-established cyclin-dependent kinase inhibitor, it is described as having an important role in controlling cell cycle progression [31]. Our results showed that STEAP1 knockdown induced p21 mRNA expression levels (2.161 ± 0.16- vs. 0.965 ± 0.026-fold variation, Figure 5b). Treatment with paclitaxel, docetaxel, or cabazitaxel also significantly increased p21 mRNA levels in scramble siRNA-transfected LNCaP cells (1.955 ± 0.05- vs. 0.965 ± 0.03-, 1.958 ± 0.03- vs. 0.965 ± 0.03-, and 2.390 ± 0.02- vs. 0.965 ± 0.03-fold variation, respectively, Figure 5b). The knockdown of the *STEAP1* gene decreased the expression of p21 induced by chemotherapeutic drugs (1.331 ± 0.08- vs. 1.955 ± 0.05-fold variation to paclitaxel, 1.174 ± 0.09- vs. 1.958 ± 0.03-fold variation to docetaxel and 1.339 ± 0.05- vs. 2.390 ± 0.02-fold variation to cabazitaxel, Figure 5b). In addition, a significant reduction in p21 expression was triggered by chemotherapeutic drugs in LNCaP cells knocked down for STEAP1 in comparison with the respective control group (Figure 5b).

### 2.5. Effect of STEAP1 Knockdown and Chemotherapeutic Drugs in Apoptosis

**Figure 6:**
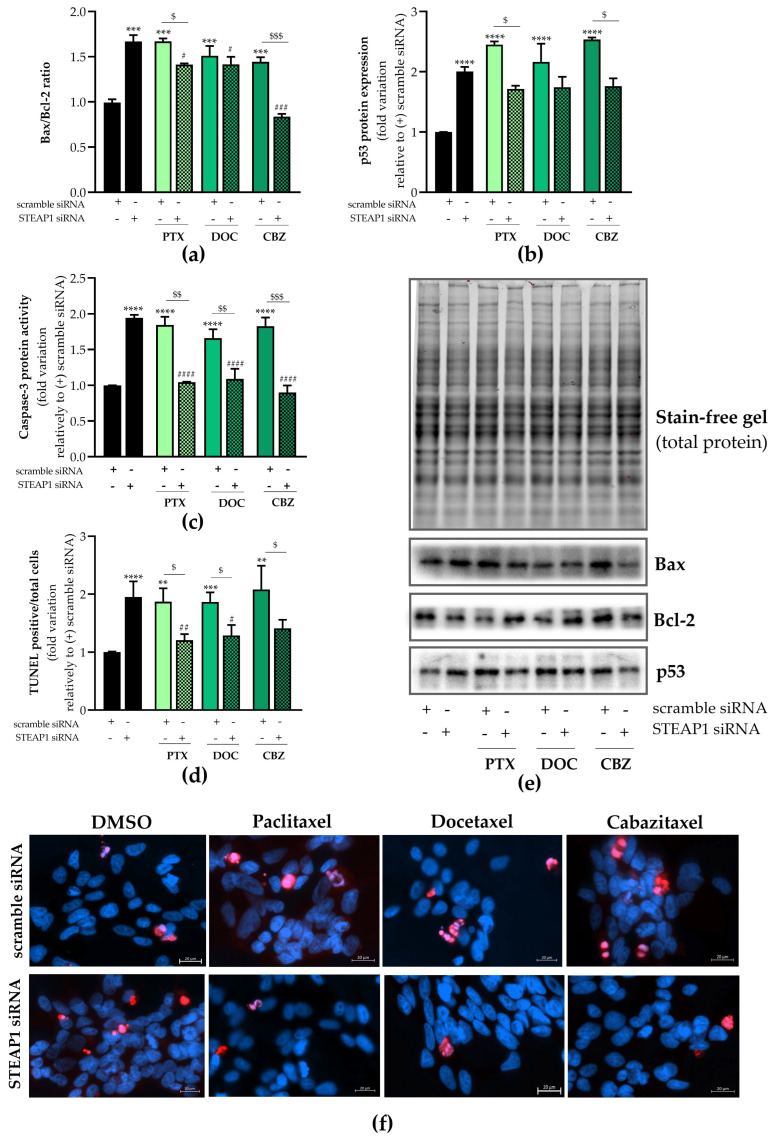
Effect STEAP1 knockdown and paclitaxel, docetaxel, or cabazitaxel on LNCaP cells apoptosis. Human neoplastic LNCaP prostate cells were transfected with scramble or STEAP1 small interfering RNA (siRNA) for 24 h and treated with 5 nM paclitaxel (PTX), 20 nM docetaxel (DOC) or 1 nM cabazitaxel (CBZ) for anadditional 24 h. The symbol “+” and “-“ means presence or absence of the type of siRNA used in each experimental group. (**a**) Bax/Bcl-2 protein ratio and (**b**) p53 protein was determined by Western blot analysis, after normalization with total protein. (**e**) Representative SDS-PAGE gels and immunoblots. (**c**) Caspase-3-like activity measured spectrophotometrically through the detection of p-NA. (**d**) TUNEL-positive LNCaP cell relatively to total cell number. (**f**) Representative fluorescent immunocytochemistry images of TUNEL-labeled cells (red) and Hoechst 33342 stained nuclei (blue) obtained in the AxioImager Z2 microscope under 400× magnification. Ten randomly selected fields per microscope cover glass were assessed. Results are shown as fold change relative to the scramble siRNA group (control). Error bars shows mean ± S.E.M (n ≥ 2). ** *p* < 0.01, *** *p* < 0.001 and **** *p* < 0.0001 when compared with the scramble siRNA group; ^#^ *p* < 0.05, ^##^ *p* < 0.01, ^###^ *p* < 0.001 and ^####^ *p* < 0.0001 when compared with the STEAP1 siRNA group; ^$^ *p* < 0.05, ^$$^ *p* < 0.01 and ^$$$^ *p* < 0.001.

The apoptotic status of LNCaP cells knocked down for STEAP1 and exposed to paclitaxel, docetaxel, and cabazitaxel was also evaluated. The results of Figure 6 showed that the pro-/antiapoptotic Bax/Bcl-2 ratio increased after STEAP1 knockdown in LNCaP cells (1.670 ± 0.04- vs. 0.993 ± 0.02-fold variation, Figure 6a). The ratio of Bax/Bcl-2 was also increased in scramble siRNA-transfected LNCaP cells treated with chemotherapeutic drugs (1.671 ± 0.02- vs. 0.993 ± 0.02-fold variation to paclitaxel, 1.512 ± 0.08- vs. 0.993 ± 0.02-fold variation to docetaxel and 1.444 ± 0.04- vs. 0.993 ± 0.02-fold variation to cabazitaxel, Figure 6a). The silencing of STEAP1 in LNCaP cells significantly reversed the effect of paclitaxel (1.413 ± 0.01- vs. 1.671 ± 0.02-fold variation, Figure 6a) and cabazitaxel (1.444 ± 0.04- vs. 0.837 ± 0.02-fold variation, Figure 6a) treatment in increasing Bax/Bcl-2 ratio. Also, a significant decrease in the Bax-Bcl-2 ratio was observed in STEAP1 siRNA LNCaP cells treated with chemotherapeutic drugs when compared to the STEAP1 siRNA group (Figure 6a). The tumor suppressor protein p53 was also evaluated. As shown in Figure 6b, a strong increase in p53 expression levels was detected in LNCaP cells silenced for STEAP1 (2.004 ± 0.08- vs. 1.001 ± 0.001-fold variation). Similar effects were seen in scramble siRNA mock-transfected LNCaP cells, upon paclitaxel, docetaxel, or cabazitaxel treatment, with the induced expression of p53 (2.452 ± 0.05- vs. 1.001 ± 0.001-, 2.164 ± 0.302- vs. 1.001 ± 0.001-, and 2.535 ± 0.04- vs. 1.001 ± 0.001-fold variation, respectively, Figure 6b). STEAP1 knockdown abolished the effect of chemotherapeutic drugs, decreasing p53 expression compared to the respective scramble siRNA drug-treated group (1.715 ± 0.05- vs. 2.452 ± 0.05-fold variation to paclitaxel, 1.744 ± 0.17- vs. 2.164 ± 0.30-fold variation to docetaxel, and 1.762 ± 0.13- vs. 2.535 ± 0.04-fold variation to cabazitaxel, Figure 6b).

Apoptosis is triggered by the caspase enzymes, and intrinsic and extrinsic pathways converge at the activation of caspase-3, which is considered a remarkable endpoint of apoptosis [32]. Caspase-3-like activity significantly increased in response to STEAP1 knockdown (~94% ± 0.04 relative to scramble siRNA, Figure 6c). With similar magnitude effects, paclitaxel, docetaxel or cabazitaxel significantly increased caspase-3-like activity in LNCaP cells knocked down for STEAP1 in comparison with the scramble siRNA control group (~86% ± 0.11, ~66% ± 0.13 and ~83% ± 0.12 increase, respectively, Figure 6c). In STEAP1-knockdown LNCaP cells, the increased effect of chemotherapeutic drugs in caspase-3-like activity was abolished (Figure 6c). Moreover, the chemotherapeutic drugs reduced the activity of caspase-3 in LNCaP cells knocked down for STEAP1 when compared to the respective control group (Figure 6c). 

Considering apoptosis based on the TUNEL assay, STEAP1-knockdown significantly increased the number of TUNEL-stained LNCaP cells compared to the scramble siRNA transfected cells (1.951 ± 0.12- vs. 1.002 ± 0.004-fold variation, Figure 6d). It was also found that in LNCaP cells transfected with scramble siRNA, the number of TUNEL-positive cells was significantly increased after treatment with paclitaxel, docetaxel, or cabazitaxel (1.872 ± 0.16- vs. 1.002 ± 0.004-fold variation, 1.867 ± 0.10- vs. 1.002 ± 0.004-fold variation and 2.081 ± 0.29- vs. 1.002 ± 0.004-fold variation, respectively, Figure 6d). The effect of chemotherapeutics was reversed when STEAP1 was knocked down in LNCaP cells (Figure 6d). Furthermore, the number of TUNEL-stained LNCaP cells was significantly decreased in cells silenced for STEAP1 and treated with paclitaxel or docetaxel drugs relative to the respective control group (Figure 6d).

## 3. Discussion

In the last few years, several pieces of evidence have associated STEAP1 with being an oncogenic protein driving the progression of several human cancers, particularly PCa [4,10,12,13,15,16]. Some strategies have been developed targeting the STEAP1 protein as a potential treatment of PCa. In fact, it has been shown that monoclonal antibodies designed against STEAP1 can inhibit PCa in mice models [2]. Our research group established that the STEAP1 knockdown reduced PCa cell growth accompanied by the enhanced rate of apoptosis [11]. One of the main strategies in cancer therapy is the use of chemotherapeutic drugs, such as paclitaxel, docetaxel, and cabazitaxel, which have emerged as the treatment of choice in PCa patients [22,26]. However, most patients develop resistance to these drugs due to changes in the expression of oncogenes [33]. Recently, it was shown that STEAP1 did not alter the response of PCa cells to anti-androgen treatment [34]. Taking into account that there are no studies evaluating the relationship between STEAP1 and taxane-based chemotherapeutics, this study intended to explore the effect of chemotherapeutic drugs in modulating the expression of STEAP1, as well as to evaluate the role of STEAP1 in influencing the response of PCa cells to chemotherapeutic drugs. 

As a first approach, it was investigated whether treatment with paclitaxel, docetaxel or cabazitaxel would modify the expression of STEAP1 protein in PCa cells (Figure 2). In LNCaP cells, it was found that both paclitaxel and cabazitaxel treatment promoted a significant increase in STEAP1 protein expression, whereas no significant effect was observed in response to docetaxel. Currently, no definitive conclusion can be drawn, but the increase in STEAP1 expression may be a way for cells to overcome the effects of paclitaxel and cabazitaxel. Curiously, the STEAP1 knockdown in LNCaP cells upon transfection with the STEAP1 siRNA was reversed in the presence of docetaxel, but not paclitaxel or cabazitaxel, which suggests that STEAP1 may also be a mediator counteracting the docetaxel effects in LNCaP cells. Regarding the C4-2B cells, no differences were observed in STEAP1 expression upon treatment with chemotherapeutic drugs. Altogether, these results suggest that the effect of chemotherapeutic drugs may be dependent on the characteristics of PCa cells. 

Next, it was analyzed whether silencing STEAP1 may affect the action of chemotherapeutic drugs in controlling the cell viability. The *STEAP1* gene silencing decreased the viability of LNCaP and C4-2B cells, as indicated by MTT assay (Figure 3). These results are in agreement with previous studies performed by our research group [11]. As expected, LNCaP and C4-2B cell viability decreased with a taxane-based drug treatment (Figure 3). Similar studies showed that treatment of PCa cells with paclitaxel, docetaxel, and cabazitaxel triggered cytotoxic effects inducing apoptosis [35,36,37]. Noteworthy, STEAP1 knockdown abolished the effect of taxane-based chemotherapeutics increasing LNCaP and C4-2B cell viability (Figure 3). In addition, it should be highlighted that these chemotherapeutic drugs increased the cell viability of PCa cells knocked down for STEAP1. These results are very interesting and are the first report indicating that the use of taxane-based drugs combined with STEAP1 knockdown may not only be ineffective but even deleterious in PCa with reduced levels of STEAP1. This was an unexpected finding since a previous study showed that the downregulation of STEAP1 significantly increased the chemosensitivity of gastric cancer cells to docetaxel treatment [38]. However, considering that the STEAP1 protein seems to act as a channel for small molecules [2], it is plausible that these drugs may also enter cells through the STEAP1 protein, or the molecules exchange across the cell membranes through the STEAP1 protein result in better uptake of these drugs (or are less extruded from the PCa cells). This hypothesis is supported by other studies involving channel proteins with a similar structure to STEAP1, such as the TRPM7 protein, which is also overexpressed in PCa and act as an oncoprotein. The knockdown of TRPM7 suppressed the migration, invasion, and proliferation of PCa cells [39,40,41]. On the other hand, the suppression of TRPM7 increased the cell viability in response to doxorubicin, indicating that reduced expression of TRPM7 may be associated with resistance to doxorubicin [42]. 

In order to deepen the knowledge underlying the role of STEAP1 and chemotherapeutic drugs in PCa progression, AKT and ERK signaling pathways were analyzed. AKT is one of the major downstream effectors of the PI3K signaling pathway to mediate cell survival, and ERK is another kinase that also regulates cell proliferation and survival of PCa cells [43,44]. The knockdown of STEAP1 decreased the levels of pAKT and pERK isoforms in PCa cells (Figure 4), which underpinned the diminished cell viability of PCa cells (Figure 3). As expected, the proliferative activity decreased in response to treatment with chemotherapeutic agents. In addition, and supporting the results obtained with MTT assay, the anti-proliferative effect of STEAP1 knockdown or drug treatment alone was abolished when both treatments were applied simultaneously. Altogether, our results indicate that these chemotherapeutic drugs may induce cell growth and proliferation in PCa cells with low levels of STEAP1. It is well-established that AKT is associated with cell survival due to the inhibition of pro-apoptotic proteins (e.g., Bax) and the activation of anti-apoptotic ones (e.g., Bcl-2) [45]. Also, the increase of the Bax/Bcl-2 ratio may induce apoptosis through the activation of caspase-3 [46]. Therefore, we have explored the role of STEAP1 and chemotherapeutic drugs in the apoptotic process. The STEAP1 knockdown or treatment with chemotherapeutic drugs significantly increased the Bax/Bcl-2 ratio and caspase-3-like activity (Figure 6), suggesting that the inhibition of apoptosis due to the overexpression of STEAP1 PCa cells may be mediated by the activation of AKT. Also, AKT signaling may be linked with the inactivation of the tumor suppressor p53 protein [47,48], which is involved in cell cycle arrest and apoptosis activation. Our results, which showed an increase in p53 expression when PCa cells were knocked down for STEAP1 or treated with chemotherapeutic drugs (Figure 6), support this connection. The role of p53 in cell cycle arrest is supported by the increased expression of p21 mRNA (Figure 5), a p53 responsive-gene that encodes an inhibitor protein of cyclin-dependent kinase at the G1 phase of the cell cycle [49]. These results are also supported by the diminished levels of pERK in response to STEAP1 knockdown or treatment with chemotherapeutic drugs. Similar results have been reported for other oncoproteins in cancer cells [50,51,52,53]. Altogether, the results obtained herein suggest that the STEAP1 knockdown and the effects of chemotherapeutic drugs in cell proliferation and apoptosis may be mediated by the MAPK and PI3K/AKT signaling pathways. However, the STEAP1 silencing combined with taxane-based drugs considerably increased the levels of pAKT in PCa cells, and that was concomitant with the increased expression of pERK levels, indicating that these drugs may cause harmful effects in PCa with a low expression of STEAP1. 

The transcription factor c-myc is essential for cell survival and proliferation and is one of the most frequently activated oncogenes, important for cancer growth and invasion [54]. Moreover, c-myc can also induce apoptosis in several cell types and appears to be a major regulator of apoptotic responses induced by a variety of stimuli, such as hypoxia, glucose deprivation, and DNA damage induced by cancer chemotherapeutics [54,55]. Noteworthy, p-c-myc expression increased in response to STEAP1 knockdown, and was also drastically increased with paclitaxel, docetaxel, and cabazitaxel (Figure 4). This increase was maintained when PCa cells were silenced for STEAP1 and treated with chemotherapeutic drugs, except for cabazitaxel where there was a significant reversal of the increase in p-c-myc expression levels. This increase in p-c-myc expression in PCa cells knocked down for STEAP1 may be a molecular mechanism to counteract the anti-proliferative action of STEAP1 knockdown. However, and considering the dual role of c-myc in cells, additional studies are required to clarify the biological significance of increased levels of c-myc upon STEAP1 knockdown in PCa cells. Nevertheless, some studies have described mechanisms that allow increased levels of c-myc in PCa due to the inhibition of ubiquitin-mediated proteasomal degradation [54,56]. This mechanism of c-myc stabilization may be linked to the dysregulation of ERK and GSK signaling, since for c-myc degradation, an initial ERK-mediated serine 62 phosphorylation is required, followed by a phosphorylation at threonine 58 by GSK-3β [56]. As ERK protein expression levels are decreased in PCa cells silenced for STEAP1, or exposed to taxane-based drugs, this may lead to an increase in c-myc protein stability. This premise is also supported by the cabazitaxel treatment, the effect of which is reversed by the knockdown of STEAP1. In the literature, there are contradictory results regarding the effect of chemotherapeutic drugs in the expression of p-c-myc. Various studies showed a down-regulation of c-myc expression in PCa cells treated with paclitaxel, docetaxel, and cabazitaxel [35,57,58]. Contrastingly, in addition to the fact that c-myc is overexpressed in CRPC and its expression is correlated with poor outcomes [59], there are studies revealing that the overexpression of c-myc in PCa cells after docetaxel treatment leads to tumorigenesis [60]. Others also showed that chronic paclitaxel treatment in metastatic CRPC cells promotes the development of resistance via upregulating c-myc expression [61]. Gathering together this information, it suggests that the overexpression of c-myc could provide conditions for the development of the resistance of taxane-based chemotherapeutics.

Overall, our results revealed that taxane-based chemotherapeutics are more effective in inducing apoptosis and suppressing viability and proliferation of PCa cells that overexpress the STEAP1 protein. Furthermore, it is important to emphasize that these chemotherapeutic drugs may have a detrimental effect on PCa cells with a decreased expression of STEAP1. These results also suggested that STEAP1 overexpression may be used as a putative positive predictive biomarker for patient selection for chemotherapy with these anti-cancer drugs. This study opens new avenues of research aiming to explore the mechanisms underlying the role of STEAP1 in human PCa. However, in order to overcome the main limitations of this study, further studies must be addressed to explore the role of STEAP1 in PCa cells treated with chemotherapeutic drugs, namely the use of genetically engineered animal models and tumor xenografts. 

## 4. Materials and Methods

### 4.1. Cell line and Culture Conditions

Human PCa cell lines (LNCaP, PC3, DU145, and 22RV1) were purchased from the European Collection of Cell Cultures (ECACC, Salisbury, UK), and C4-2B and VCaP cell lines were kindly provided by Professor Carmen Jerónimo from Cancer Biology & Epigenetics Group, Research Center of IPO Porto. All the cell lines were maintained in RPMI-1640 phenol-red medium (Sigma Aldrich, St. Louis, MO, USA) supplemented with 10% fetal bovine serum (FBS, Biochrom AG, Berlin, Germany) and 1% penicillin/streptomycin (Gibco, Grand Island, NE, USA), at 37 °C in an atmosphere equilibrated with 5% CO_2_.

### 4.2. Small-Interfering RNA Transfection and Treatments

At 50% confluence, LNCaP and C4-2B cells were transfected with 20 nM of a small interfering RNA (siRNA) targeting STEAP1 (STEAP1 siRNA, s226093, Ambion, Carlsbad, CA, USA), or scramble siRNA (s4390846, Ambion) for 24 h. For this purpose, the appropriate quantity of scramble- and STEAP1 siRNA was diluted in Opti-MEM^®^ (mix A). Simultaneously, Lipofectamine 3000 (Invitrogen, Carlsbad, CA, USA) was diluted in Opti-MEM^®^ (mix B), according to the manufacturer’s instructions. After incubation for 5 min at room temperature (RT), mix A and B were combined, and the formation of siRNA: Lipofectamine complexes occurred for additional 20 min at RT. Then, the complexes were added to cells. After 24 h of transfection, cells were treated with chemotherapeutics drugs, 5nM of paclitaxel (Alfa Aesar, Haverhill, MA, USA), 20 nM of docetaxel (Sigma Aldrich, St. Louis, MO, USA) and 1nM of cabazitaxel (Sigma Aldrich, St. Louis, MO, USA), for additional 24 h. All drugs were prepared with DMSO and concentrations were selected according to the literature [62,63,64]. The efficiency of STEAP1 expression knockdown was evaluated by quantitative real-time PCR (qPCR) and Western blot.

### 4.3. Reverse Transcription Real-Time Quantitative Polymerase Chain Reaction (RT-qPCR)

Total RNA was isolated from LNCaP and C4-2B cells using TRI reagent (Grisp, Lisboa, Portugal) in accordance with the manufacturer’s protocol. Total RNA was quantified by spectrophotometry at 260 and 280 nm (Pharmacia Biotech, Ultrospec 3000, Denmark), and its integrity using an agarose gel electrophoresis. 200 ng of total RNA was used for cDNA synthesis and the expression of STEAP1 and p21 genes was determined using Power SYBR Green RNA-to-CT, 1-Step Kit (Applied Biosystems, Waltham, MA, USA) and the CFX connect real-time system (Bio-Rad, Hercules, CA, USA). RT-qPCRs were performed in a final volume of 10 µL with STEAP1 (sense: 5′ GGC GAT CCT ACA GAT ACA AGT TGC 3′ and anti-sense: 5′ CCA ATC CCA CAA TTC CCA GAG AC 3′), p21 (sense: 5′ TCC AGC GAC CTT CCT CAT C 3′ and anti-sense: 5′ AGC CTC TAC TGC CAC CAT C 3′), and β-2-microglobulin (β2M) (sense: 5′ ATG AGT ATG CCT GCC GTG TG 3′ and anti-sense: 5′ CAA ACC TCC ATG ATG CTG CTTAC 3′) specific primers. Annealing temperature was 60 °C for all primer sets and samples were run in triplicate for three independent experiments. β2M housekeeping was used as an internal control to normalize gene expression. Normalized expression values were calculated following the model proposed by Pfaffl [65] using the formula: 2^−∆∆Ct^.

### 4.4. Protein Extraction and Western Blot

LNCaP and C4-2B cells were homogenized in radioimmunoprecipitation assay buffer (RIPA, 150 mM NaCl, 1% Nonidet-P40, 0.5% Na-deoxycholate, 0.1% SDS, 50 mM Tris) supplemented with 10% PMSF and 1% protease inhibitors cocktail, kept on ice for 30 min with occasional vortex. Next, the homogenized was centrifuged at 14,000 rpm for 20 min at 4 °C. Total protein was quantified using the Pierce 660 nm Protein assay reagent (Thermo Scientific, Waltham, MA, USA). A total of 20 µg of total protein was resolved on 10% TGX Stain-free polyacrylamide gels (Bio-Rad), scanned in the ChemiDoc™ MP Imaging System (Bio-Rad), and then electrotransferred to a PVDF membrane (BioRad). After blocking with 5 % milk solution, membranes were incubated with rabbit anti-STEAP1 (1:1000, D8B2V, Cell Signaling Technology, Danvers, Massachusetts, EUA), rabbit anti-pAKT (1:500, ref. #9271, Cell Signaling Technology), rabbit anti-pERK (1:500, ref. #9101, Cell Signaling Technology), rabbit anti-p-c-myc (1:500, ref. #13748, Cell Signaling Technology), rabbit anti-Bcl-2 (1:1000, ref. #2876, Cell Signaling Technology), rabbit anti-Bax (1:1000, ref. #2772, Cell Signaling Technology), and rabbit anti-p53 (1:1000, FL-393:sc-6243, Santa Cruz Biotechnology) overnight at 4°C. Anti-rabbit IgG-HRP (1:15000, Sigma-Aldrich) was used as a secondary antibody. Membranes were incubated with the ECL substrate (Bio-Rad) and scanned using the ChemiDoc™ MP Imaging System (Bio-Rad). Band densities were obtained by densitometry analysis using the Image Lab 5.1 software (Bio-Rad) and normalized to the total protein on the gel [66].

### 4.5. Cell Viability Assay

STEAP1 siRNA- and scramble siRNA-transfected cells (25,000 cells/well) that were placed for 24 h and treated with paclitaxel, docetaxel and cabazitaxel drugs (24 h) were grown in 96-well plates, and cell viability was determined by the colorimetric MTT assay. In brief, MTT (3-(4,5-Dimethyl-2-thiazolyl)-2,5-diphenyl-2H-tetrazolium bromide), at 0.5 mg/mL final concentration, was added to the cell culture medium and the reaction occurred in the dark at 37 °C for 1 h. Next, the MTT solution was carefully removed and the formazan crystals were solubilized with 100 µL of DMSO. The absorbance of the solution was determined at 570 nm using the xMark™ Microplate Absorbance Spectrophotometer (Bio-Rad). The absorbance value is proportional to the number of viable cells in each experimental group.

### 4.6. Ki-67 Fluorescent Immunocytochemistry

Cells were fixed with 4% paraformaldehyde and permeabilized with 1% triton X-100 for 5 min at RT. In order to block non-specific binding sites, the cells were incubated with PBS containing 0.1% (*w/v*) Tween-20 and 20% FBS for 1 h. After washing, cells were incubated for 1 h at RT with rabbit anti-Ki-67 (1:50, n°16667, Abcam) and incubated with the Alexa Fluor 546 goat anti-rabbit IgG (1:1000, Invitrogen) secondary antibody for 1 h at RT. Cells were washed with PBS and stained with Hoechst-33342 (5 µg/mL, Invitrogen) for 5 min. Then, the coverslips were rinsed with PBS, mounted using Dako (Invitrogen) and visualized by fluorescence microscopy (Zeiss AxioImager A1, Carl Zeiss, Germany). The index of proliferation was determined by counting the number of Ki-67-staining cells and Hoechst-stained nuclei in ten randomly selected 40× magnification fields for each coverslip. The ratio between the number of Ki-67-stained cells and the total number of nuclei was determined.

### 4.7. Terminal Deoxynucleotidyl Transferase-Mediated dUTP Nick End Labeling (TUNEL) Assay

TUNEL analysis was performed using the In Situ Cell Death Detection Kit, TMR red kit (Roche, Mannheim, Germany) following the manufacturer’s instructions. Briefly, cells were fixed with 4% paraformaldehyde for 30 min at RT and then permeabilized with 1% Triton X-100 in phosphate buffer saline (PBS-T). Cells were stained with TUNEL reaction mixture for 1 h at RT in the dark. After washing in PBS-T, cell nuclei were stained with Hoechst-33342 (5 µg/mL, Invitrogen) for 5 min. Coverslips were mounted in Dako mounting medium (Invitrogen) and fluorescence microscopy images were acquired using a Zeiss AxioImager A1 microscope. The apoptotic index was determined by counting the number of TUNEL-positive cells and Hoechst-stained nuclei in ten randomly selected 40× magnification fields for each coverslip.

### 4.8. Caspase-3-like Activity Assay

The caspase-3-like activity was determined spectrophotometrically by detecting the presence of the yellow product p-nitroaniline (p-NA), upon cleavage of caspase-3-substrate (Ac-DEVD-p-NA). A total of 25 µg of total protein extract was incubated with a reaction buffer (25 mM HEPES, pH 7.5, 0.1% CHAPS, 10% sucrose, and 10 mM DTT) and 2 mM Ac-DEVD-p-NA. The reaction was left to occur for two h at 37 °C and the absorbance was measured at 405 nm using the xMark™ Microplate Absorbance Spectrophotometer (Bio-Rad). The amount of generated p-NA was calculated by extrapolation with a standard curve with known concentrations of p-NA.

### 4.9. Statistical Analysis

The statistical significance of all experimental groups was assessed by Student’s t-test or by ANOVA followed by Sidak’s multiple comparisons test. Significant differences were considered when *p* < 0.05 (*, ^#^), *p* < 0.01 (**, ^##^), *p* < 0.001 (***, ^###^) and *p* < 0.0001 (***, ^####^). All experimental data are shown as mean ± S.E.M. The Graphpad Prism 7.01 program (GraphPad Software, Boston, MA, USA) was used for this analysis.

## 5. Conclusions

Taxane-based chemotherapeutics have distinct effects on PCa cells depending on STEAP1 protein expression levels. Moreover, it was addressed for the first time the effect of these drugs in combination with the overexpression or knockdown of STEAP1 oncoprotein expression in PCa cells. It allowed to conclude that the use of paclitaxel, docetaxel, and cabazitaxel is more effective in PCa cells that overexpress the STEAP1 protein. Although further studies are required, it is worth noting that PCa cells with reduced expression of STEAP1 may cause harmful effects in response to chemotherapeutic drugs. 

## Data Availability

Not applicable.
